# No impact of prenatal paracetamol and folic acid exposure on cord blood DNA methylation in children with attention-deficit/hyperactivity disorder

**DOI:** 10.3389/fgene.2023.1204879

**Published:** 2023-06-15

**Authors:** Emilie Willoch Olstad, Hedvig Marie Egeland Nordeng, Robert Lyle, Kristina Gervin

**Affiliations:** ^1^ Pharmacoepidemiology and Drug Safety Research Group, Department of Pharmacy, Faculty of Mathematics and Natural Sciences, University of Oslo, Oslo, Norway; ^2^ PharmaTox Strategic Research Initiative, Faculty of Mathematics and Natural Sciences, University of Oslo, Oslo, Norway; ^3^Department of Child Health and Development, Norwegian Institute of Public Health, Oslo, Norway; ^4^Department of Medical Genetics, Oslo University Hospital and University of Oslo, Oslo, Norway; ^5^ Centre for Fertility and Health, Norwegian Institute of Public Health, Oslo, Norway; ^6^Department of Research and Innovation, Division of Clinical Neuroscience, Oslo University Hospital, Oslo, Norway

**Keywords:** ADHD (attention deficit and hyperactivity disorder), DNA methyaltion, EWAS, epigenome wide association study, folic acid (FA), MoBa (norwegian mother and child cohort study), paracetamol, MBRN

## Abstract

Pharmacoepigenetic studies are important to understand the mechanisms through which medications influence the developing fetus. For instance, we and others have reported associations between prenatal paracetamol exposure and offspring DNA methylation (DNAm). Additionally, folic acid (FA) intake during pregnancy has been associated with DNAm in genes linked to developmental abnormalities. In this study, we aimed to: (i) expand on our previous findings showing differential DNAm associated with long-term prenatal paracetamol exposure in offspring with attention-deficit/hyperactivity disorder (ADHD), and (ii) examine if there is an interaction effect of FA and paracetamol on DNAm in children with ADHD. We used data from the Norwegian Mother, Father and Child Cohort Study (MoBa) and the Medical Birth Registry of Norway (MBRN). We did not identify any impact of paracetamol or any interaction effect of paracetamol and FA on cord blood DNAm in children with ADHD. Our results contribute to the growing literature on prenatal pharmacoepigenetics, but should be replicated in other cohorts. Replication of pharmacoepigenetic studies is essential to ensure robust findings and to increase the clinical relevance of such studies.

## 1 Introduction

There is an increasing interest in understanding how maternal medication use during pregnancy may affect epigenetic patterns and impact fetal development (Olstad et al., 2021). Epigenetics entails modifications to the DNA which may alter gene expression, without changing the DNA sequence (Rakyan et al., 2011). DNA methylation (DNAm) is the most commonly studied epigenetic modification, whereby a methyl group is attached to cytosine-phosphate-guanine dinucleotides (CpGs) (Rakyan et al., 2011). DNAm is reversible and influenced by both genetics and environmental factors, such as medications (Rakyan et al., 2011; Olstad et al., 2021). The timely establishment of epigenetic patterns is pivotal for normal embryonic and fetal development (Slieker et al., 2015; Luo et al., 2018). During this time, the epigenome is highly plastic, to accommodate the complex process of cellular differentiation, essential to develop functioning organs such as the brain (Slieker et al., 2015; Ciernia and LaSalle, 2016; Luo et al., 2018). Consequently, in fetal life, the epigenome is particularly susceptible to environmental influences, which may contribute to deviations in the normal epigenetic orchestration of cellular differentiation. To this end, pharmacoepigenetic studies are useful to better understand the mechanisms through which medications may impact the developing fetus.

Epidemiological studies have suggested adverse neurodevelopmental outcomes of long-term paracetamol use during pregnancy, including increased risks of attention-deficit/hyperactivity disorder (ADHD) and autism spectrum disorder (ASD) (Bauer et al., 2021). In contrast, folic acid (FA) intake during pregnancy has been associated with reduced ADHD and ASD symptoms in the child (Li et al., 2019). Furthermore, maternal FA intake in pregnancy has been found to reduce the negative impact of prenatal antiepileptic medication exposure on verbal abilities in children at both 1.5, 3, 5, and 8 years old (Husebye et al., 2018; Husebye et al., 2020). These findings suggest FA as a moderator of the association between prenatal medication exposure and child verbal abilities. FA is an essential methyl donor for DNAm. Interestingly, a meta-analysis (Joubert et al., 2016) and a recent FA intervention study (Ondičová et al., 2022), found an association of maternal FA intake during pregnancy with differences in DNAm at genes linked to developmental abnormalities. Several studies have also found associations between prenatal paracetamol exposure and differential DNAm in cord blood (Gervin et al., 2017; Eslamimehr et al., 2022) and placenta (Addo et al., 2019), but none of the differences in DNAm overlapped across the studies (Gervin et al., 2017; Addo et al., 2019; Olstad et al., 2021; Eslamimehr et al., 2022).

Based on these pharmacoepidemiological and -epigenetic findings, we postulated that DNAm could be a plausible biological mechanism of action for the paracetamol-ADHD associations (Gervin et al., 2017). Therefore, we have previously conducted an epigenome-wide association study (EWAS) on the association of prenatal paracetamol exposure with DNAm in children with ADHD (Gervin et al., 2017). In our previous study, we found that DNAm at more than 6,000 CpGs was associated with prenatal paracetamol exposure only in children later developing ADHD (Gervin et al., 2017). To strengthen these findings, we wanted to replicate and expand on our previous study, both by increasing the number of samples and CpGs, and by exploring whether FA may impact a potential effect of paracetamol on DNAm. To this end, we aimed to: (i) expand on our previous findings showing epigenome-wide differences in DNAm associated with long-term prenatal paracetamol exposure (≥20 days) in children with ADHD (Gervin et al., 2017), and (ii) examine if there is an interaction effect of FA and paracetamol on DNAm in children with ADHD. To do this, we selected umbilical cord blood samples from the Norwegian Mother, Father and Child Cohort Study (MoBa), which contains information on maternal use of paracetamol and FA during pregnancy, and conducted an EWAS.

## 2 Materials and methods

This study is based on umbilical cord blood samples from MoBa, conducted by the Norwegian Institute of Public Health (NIPH) (Magnus et al., 2006; Magnus et al., 2016). MoBa is a prospective, population-based birth cohort (*n* = 114,500 children, *n* = 95,200 mothers and *n* = 75,200 fathers), including births in Norway between 1998 and 2008 (Magnus et al., 2006; Magnus et al., 2016). In 40.6% of pregnancies, parents consented to participate. Throughout pregnancy and childhood, the participants complete multiple questionnaires. MoBa also includes a biobank with approximately 90,000 blood samples collected from both parents during pregnancy, and from mother and child (umbilical cord) at birth (Paltiel et al., 2014). This study is based on Data Version 8 released by MoBa in 2015. All MoBa participants have provided a written, informed consent to participate in the cohort and can retract their consent at any time. The establishment of MoBa and initial data collection was based on a license from the Norwegian Data Protection Agency and approval from The Regional Committees for Medical and Health Research Ethics. The MoBa cohort is currently regulated by the Norwegian Health Registry Act. All the analyzed data were de-identified, and the linking of MoBa to the relevant health registries was handled by NIPH and the respective registries. Our study was approved by the Regional Committee for Medical Research Ethics South East Norway.

The selection of individuals for the study was based on observational data from MoBa questionnaires Q1 (gestational weeks 0–13), Q3 (gestational weeks 13–29) and Q4 (gestational week 30 to delivery), the Medical Birth Registry of Norway (MBRN) and the Norwegian Patient Registry (NPR). MBRN is a national health registry containing information about all births in Norway and NPR contains diagnoses asserted by specialists in governmental hospitals and outpatient clinics. MoBa was linked to NPR and MBRN using the personal 11-digit identification number unique to every permanent resident of Norway. We included 261 pregnancies from MoBa, divided into three groups: (i) children with ADHD prenatally exposed to paracetamol ≥20 days (exposed group; *n* = 61), (ii) children with ADHD and unexposed to paracetamol (ADHD-controls; *n* = 100), and (iii) children without ADHD and unexposed to paracetamol (population controls; *n* = 100). We did not include a paracetamol-control group (i.e., children prenatally exposed to paracetamol ≥20 days without ADHD), as in our prior study we did not identify any significant differences in DNAm between this group and the exposed group (Gervin et al., 2017). Pre-term births (<37 weeks) and twins were excluded. The definitions of the different measures and the selection of covariates are presented in the Supplementary Methods (Supplementary Methods).

DNAm was assessed using the Infinium HumanMethylation EPIC BeadChip (Illumina). Samples were randomly allocated to sample plates and beadchips, and processed as previously described (Gervin et al., 2017). All analyses were performed in the R programming language (http://www.r-project.org/). The DNAm data was quality controlled using the quality control module implemented in RnBeads (v. 2.8.1) (Assenov et al., 2014; Müller et al., 2019). First, samples with >5% low-quality CpGs or low bisulfite intensity were removed (0 samples). CpGs with >5% low-quality values were also removed (*n* = 8,947). Low-quality probes either exhibited a detection *p*-value > 10^−6^ or a bead count <3. We performed background correction with the ENmix exponential-truncated-normal out-of-band (oob) method (Xu et al., 2016), dye bias correction with RELIC (REgression on Logarithm of Internal Control probes) (Xu et al., 2017) and probe-type correction with RCP (Regression of Correlated Probes) (Niu et al., 2016). We then removed probes with SNPs overlapping with the CpG interrogation site or the nucleotide extension site (*n* = 29,176), cross-reactive probes (*n* = 14,921) (McCartney et al., 2016; Pidsley et al., 2016) and probes on the sex chromosomes (*n* = 17,532). The final data set included 795,515 probes and 261 samples.

We used *β* values (ratio of methylated signal to total signal) for visualizations and *M* values for statistical tests (Du et al., 2010). Principal component analysis (PCA) on the DNAm data was used to test the strength of association between covariates and DNAm variation (tests applied on categorical and continuous variables included one-way analysis of variance [ANOVA] and Spearman’s correlation test, respectively). To identify differentially methylated CpGs associated with paracetamol, we fit linear regression models onto the mean DNAm differences in *limma* (Smyth et al., 2005). We included the CD8^+^ T cell proportion as a covariate in all models. Interaction was assessed by including an interaction term of paracetamol and FA in the model. We pairwise compared the exposed group to the ADHD-control and the population control groups. All comparisons were adjusted for multiple testing (false discovery rate [FDR] <0.05) (Benjamini, 2010). We performed a surrogate variable analysis to examine unmeasured sources of variation in DNAm (Leek et al., 2012). Further details on the statistical analyses are presented in the Supplementary methods (Supplementary Methods).

## 3 Results

To enable a systematic replication and expansion of our previous findings (Gervin et al., 2017), we selected three groups: one *exposed group*, consisting of children with ADHD prenatally exposed to paracetamol ≥20 days (*n* = 61), and two control groups, the *ADHD-control group*, including children with ADHD and unexposed to paracetamol (*n* = 100), and the *population control group*, including children without ADHD and unexposed to paracetamol (*n* = 100). The samples were selected from MoBa, the same cohort as our previous study was based on, and 17 of the 19 exposed samples in the original study were also included in the present study. We selected model covariates by assessing their contribution to DNAm variation using PCA (Supplementary Methods). These analyses identified that the estimated CD8^+^ T cell proportion was significantly associated with principal components (PCs) 2 and 3, and differed between the comparison groups (Supplementary Figure S1; Supplementary Tables S1, S2), and therefore, this covariate was included in the regression models.

We ran three different models to assess the impact of paracetamol and FA on DNAm in children with ADHD (Table 1): (i) a *crude model* to examine whether prenatal paracetamol exposure was associated with DNAm in the cord blood of children with ADHD, (ii) an *adjusted model* where we adjusted the crude model for FA intake during pregnancy to investigate the influence of FA status on the variance in DNAm, and (iii) an *interaction model* where we examined any interaction effect of FA and paracetamol on DNAm. We ran the three models comparing the exposed group to each of the two control groups separately, and also ran sensitivity analyses excluding the CD8^+^ T cell proportion from the models, which did not change the results (Supplementary Figure S2).

**TABLE 1:** Overview of the three models used to assess the association of prenatal paracetamol and folic acid (FA) exposure with cord blood DNA methylation (DNAm) in children with attention-deficit/hyperactivity disorder (ADHD). We ran each model separately for the exposed group compared to each of the two control groups.

Model^a^	Definition	Group comparisons	Purpose
Crude^b^ model	DNAm ∼ group + CD8^+^ T cells	**Comparison 1** Exposed vs. ADHD-control	Replicate our previous results Gervin et al. (2017) in a different study population from MoBa
		**Comparison 2** Exposed vs. population control	
Adjusted model	DNAm ∼ group + FA + CD8^+^ T cells	**Comparison 1** Exposed vs. ADHD-control	Address the influence of FA intake on the variance in DNAm
		**Comparison 2** Exposed vs. population control	
Interaction model	DNAm ∼ group*FA + CD8^+^ T cells	**Comparison 1** Exposed vs. ADHD-control	Assess whether there is an interaction effect of paracetamol and FA intake on DNAm
		**Comparison 2** Exposed vs. population control	

Abbreviations: ADHD, attention-deficit/hyperactivity disorder; DNAm, DNA, methylation; FA, folic acid; MoBa, the Norwegian Mother, Father and Child Cohort Study.

^a^ Sensitivity analysis excluding CD8^+^ T cell proportion from the model produced similar results.

^b^ Crude with respect to folic acid intake.

**FIGURE 1:**
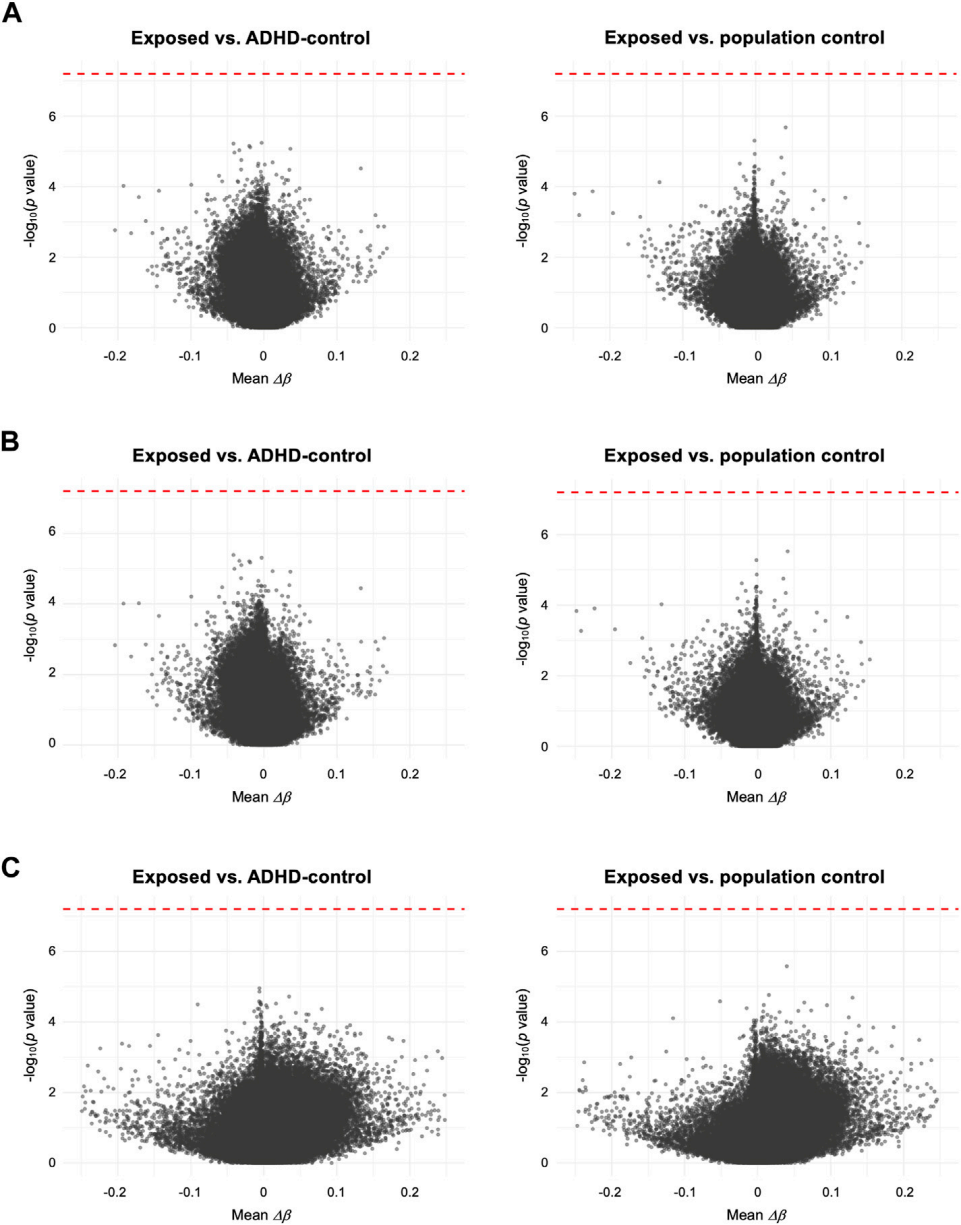
Volcano plots comparing mean differences in DNAm (*Δβ*) for each CpG between the exposed group and either the ADHD-control group or the population control group. **(A)** Crude model, only adjusted for CD8^+^ T cell proportion. **(B)** Adjusted model, adjusting for FA intake during pregnancy and CD8^+^ T cell proportion. **(C)** Interaction model, assessing any interaction effect of prenatal paracetamol and FA exposure on DNAm. Each CpG is plotted by the -log_10_ of the *p* values against the mean per-CpG difference in DNAm (*Δβ*) of the compared groups. In **(C)** mean *Δβ* reflects the interaction term (i.e., mean *Δβ*_no FA_–*Δβ*_FA_). The red dotted line indicates the FDR-adjusted *p* value significance threshold (FDR < 0.05)*.*

To test the association of DNAm with prenatal paracetamol exposure and ADHD, we ran linear regression models comparing the exposed group to the ADHD-control group and the population control group (the crude model). This analysis did not identify any significant differences between the exposed group and either of the two control groups (Figure 1A; Supplementary Figure S3A; Supplementary Table S3), and thereby did not replicate our previous findings. We then assessed whether FA could influence the association between paracetamol exposure and DNAm by running an adjusted model, adjusting for FA intake during pregnancy. This analysis did not reveal any significant CpGs and the *p* values were largely similar to those observed in the crude model (Figure 1B; Supplementary Figure S3B). Finally, to understand whether there is an interaction effect of paracetamol and FA intake on DNAm in children with ADHD, we ran an interaction model where the group and FA exposure variables interacted. This analysis neither revealed any significant CpGs (Figure 1C; Supplementary Figure S3C).

For the crude model, we also explored whether the difference in results between our present and previous study could be explained by differences in normalization methods, selection of covariates or number of probes on the microarrays (EPIC vs. 450 k, respectively). Conducting our analysis using the same normalization methods (i.e., noob and BMIQ) and/or the same covariates as in our previous study (i.e., sex, smoking, maternal age, gestational age, estimated cell type composition, BeadChip and bisulfite conversion plate), we still find no significant CpGs (Supplementary Table S4). Yet, some CpGs exhibit FDR-adjusted *p* values closer to significance (*p* ≥ 0.13), but none of these overlap with the significant findings in our previous study. Finally, to assess whether the heightened burden of multiple testing associated with the increased number of probes on the EPIC microarray could explain our null findings, we also ran the crude model only on the probes overlapping between the 450 k and EPIC microarrays (Supplementary Table S5). This analysis did not reveal any CpGs significantly associated with prenatal paracetamol exposure in children with ADHD.

## 4 Discussion

Overall, we identified no impact of paracetamol nor interaction of paracetamol and FA on cord blood DNAm in children with ADHD in our data. Results from the crude model did not identify any significant differences in DNAm between the exposed group and either of the two control groups. Consequently, we did not replicate our previous results (Gervin et al., 2017). These findings were surprising, as the present study is based on a larger number of samples from the same cohort (MoBa), using the same study design and similar inclusion criteria as in the original study. However, our studies are performed 5 years apart and methods have evolved, including the introduction of the Illumina Infinium EPIC platform and novel analysis methods, such as normalization and cell type deconvolution procedures. While we could not explain our replication failure solely by differing DNAm measurements between platforms (Olstad et al., 2022), we cannot exclude that other aspects of the analysis may have contributed to the lack of replication.

To explore if differences in the analysis between our present and previous study contributed to our non-replication, we examined whether varying normalization methods, covariates and number of probes on the microarrays would change the results. We did not identify any CpGs significantly associated with prenatal paracetamol exposure in children with ADHD in these analyses.

Our findings also differ from two recent studies on prenatal paracetamol exposure and DNAm, which found differential DNAm at some CpGs associated with paracetamol (Addo et al., 2019; Eslamimehr et al., 2022). However, comparison to these studies is difficult for several reasons, including differing study designs, exposure definitions, phenotypes, tissue types and analysis pipelines. There are also other challenges when comparing current prenatal pharmacoepigenetic studies (Olstad et al., 2021), which may contribute to the lack of replication and overlap of findings.

When assessing whether FA contributed to the variance in DNAm or if there was an interaction effect of paracetamol and FA on DNAm, we did not find any significant CpGs, suggesting that there is no effect of paracetamol and FA intake on DNAm in children with ADHD. Previous studies have reported differential DNAm associated with maternal FA intake during pregnancy in genes linked to developmental abnormalities (Joubert et al., 2016; Ondičová et al., 2022). However, these studies examined FA intake as the main exposure rather than as an interacting factor with medication use (Joubert et al., 2016; Ondičová et al., 2022), and therefore, are not directly comparable to the current study.

While we have improved multiple aspects of our previous study (Gervin et al., 2017), there are some limitations to the current study. The sample sizes of the groups are relatively small, albeit larger than in our previous study. Although the MoBa cohort is one of the world’s largest prospective birth cohorts, only 61 children with a clinical ADHD diagnosis were exposed to paracetamol ≥20 days. Additionally, we did not assess the dose and timing of FA intake during pregnancy. However, in Norway, the recommended FA supplement dosage is 400 *μ*g/day, starting 1 month prior to pregnancy and lasting throughout the first trimester of pregnancy.

In conclusion, this study did not replicate previous findings in MoBa or other studies investigating the influence of paracetamol on DNAm, and did not identify any interaction effect of paracetamol and FA on DNAm in children with ADHD. Our results contribute to the growing literature on prenatal pharmacoepigenetics, but should be replicated in other cohorts. Replication of pharmacoepigenetic studies is essential to ensure robust findings and to increase the clinical relevance of such studies.

## Data Availability

The datasets presented in this article are available from the Norwegian Mother, Father and Child Cohort Study, but restrictions apply to the availability of these data and so are not publicly available. However, data are available from the authors upon reasonable request and with permission from the Norwegian Mother, Father and Child Cohort Study. Requests to access the datasets should be directed to EWO, e.w.olstad@farmasi.uio.no.
