# Supplementary material for: No impact of prenatal paracetamol and folic acid exposure on cord blood DNA methylation in children with attention-deficit/hyperactivity disorder
Source: Front Genet. 2023 Jun 15;14:1204879. doi: 10.3389/fgene.2023.1204879 (PMC10310527; doi:10.3389/fgene.2023.1204879)
Supplement: Supplementary file 1 [file DataSheet1.docx]

Supplementary Material

**No impact of prenatal paracetamol and folic acid exposure on cord blood DNA methylation in children with attention-deficit/hyperactivity disorder**

Emilie Willoch Olstad^*^, Hedvig Marie Egeland Nordeng, Robert Lyle, Kristina Gervin

*** Correspondence:** Emilie Willoch Olstad: [e.w.olstad@farmasi.uio.no](mailto:e.w.olstad@farmasi.uio.no)

# Supplementary Methods

## Paracetamol exposure definition

# Paracetamol use was retrieved from self-reports in three Mother, Father and Child cohort study questionnaires (MoBa; Q1, Q3, and Q4). Pregnant people report use of paracetamol per every 4-week interval of their pregnancy, in questionnaires distributed during and after pregnancy. Q1 and Q3 are distributed around gestational weeks 15 (0–4; 5–8; 9–12; ≥13 weeks) and 30 (13–16; 17–20; 21–24; 25–28; ≥29 weeks), respectively. Q4 is distributed approximately 6 months after delivery and covers the last weeks of pregnancy (from gestational week 30 until delivery). Women were presented with a number of illnesses and problems, and could report any medications used for the respective indications. The women also reported the total number of days they used the medication for each specific indication. We defined long-term prenatal exposure to paracetamol (Anatomical Therapeutic Chemical [ATC] code: N02BE01), as the use of paracetamol for ≥20 days during pregnancy (any indication), as in our previous study [1]. Paracetamol exposure was coded as a binary “yes”/“no” variable.

## Attention-deficit/hyperactivity disorder definition

# We retrieved offspring attention-deficit/hyperactivity disorder (ADHD)-diagnosis from the Norwegian Patient Registry (NPR; 2008–2016), which contains all diagnoses asserted by specialists in governmental hospitals and outpatient clinics, in accordance with the 10th revision of the International Classification of Disease (ICD-10). ADHD was defined as an ICD-10 diagnosis of hyperkinetic disorder (HKD; F90.0, F90.1, F90.8, or F90.9) between 2008 and 2016. HKD corresponds to ADHD in the Diagnostic and Statistical Manual (DSM) system [2–5].

## Folic acid exposure definition

# Folic acid (FA) use is recorded in both MoBa and the medical birth registry of Norway (MBRN). In MoBa, pregnant people report use of FA per every 4-week interval of their pregnancy, in questionnaires Q1 and Q3 described above. Self-reported FA use in MoBa corresponds well with maternal FA serum concentrations at 19 weeks of pregnancy [6, 7]. In MBRN, use of FA is recorded upon interview during the first antenatal consultation (pregnancy weeks 6–12). In this consultation, the pregnant woman is asked whether she has used FA prior to and/or during pregnancy. If she answers “yes”, this is recorded as FA use in the corresponding check box (FA prior to pregnancy and/or FA during pregnancy). We defined exposure to FA in pregnancy as pregnant people with recordings of FA use during pregnancy in both MoBa and MBRN (*n*_FA_ = 135; *n*_no FA_ = 126).

## Covariates

# Analyses of potential covariates for inclusion in the linear regression models were done in three steps. First, we performed a principal component analysis (PCA) to analyze the association between the first three principal components (PCs), explaining the most DNAm variation across samples, and relevant covariates (Figure S1A–B). We assessed the relevant covariates previously included in prenatal pharmacoepigenetic studies [8]: maternal age, maternal education, marital status, primiparity, alcohol use, smoking, multivitamin use, psychotropic and analgesic use, maternal diseases, in vitro fertilization, Caesarean section, gestational age, infant sex, birth weight, bisulfite conversion and the composition of all cell types (CD8^+^ T cells, CD4^+^ T cells, natural killer cells, B cells, monocytes, granulocytes and nuclear red blood cells [nRBCs]). Second, we assessed the individual contribution of the significant covariates to the variation in DNAm (Figure S1C), as described elsewhere [9, 10]. Briefly, we fit multivariable linear regression models for each of the first three PCs. The explanatory variables were the covariates associated with either of the three PCs. For each PC, we found the partial R^2^, indicating the variation each covariate contributed to the variation of the PC, when accounting for the contribution of all other covariates in the model. The weighted partial R^2^ for the three PC models was reported as the variability contributed to DNAm variation by the respective covariate. We found that all covariates except bisulfite conversion and cell type composition contributed <0.1% of the variation in DNAm. Finally, we tested whether the covariates contributing the most to the DNAm significantly differed between the comparison groups (Tables S1–2), resulting in a model including only the CD8^+^ T cell proportion as a covariate.

# We estimated cell type composition (CD8^+^ and CD4^+^ T cells, natural killer cells, B cells, monocytes, granulocytes and nucleated red blood cells [nRBCs]), using the “estimateCellCounts2” function of minfi [11] (Table S2). This function deconvolves the data using the Houseman reference-based approach [12] and depends on a recently published, validated cord blood reference data set (FlowSorted.CordBloodCombined.450k) [13, 14].

## Statistical analyses

### Hypothesis tests

# Significant differences of covariates between groups were tested using the Wilcoxon’s rank-sum test (continuous variables), and Chi-squared test or Fisher’s exact test (categorical variables), as appropriate. To test which covariates were significantly associated with the most DNAm variation, we used Spearman’s correlation test (continuous variables) and one-way analysis of variance (ANOVA; categorical variables).

### Differential DNA methylation analyses

# The *β* values (the ratio of methylated signal to the sum of methylated and unmethylated signal) were used for visualisation purposes, while *M* values (log_2_(*β* /(1– *β*)) were used for statistical tests, as recommended based on their statistical properties [15]. To identify differentially methylated sites associated with paracetamol, we fit linear regression models onto the mean DNAm differences, using limma [16]. Interaction was assessed by including an interaction term in the model. We pairwise compared the exposed group to the ADHD-control and the population control groups. All comparisons were adjusted for multiple testing with a false discovery rate (FDR) cut off <0.05, using the Benjamini and Hochberg method [17].

### Surrogate variable analysis

# We performed a surrogate variable analysis to examine any unmeasured sources of variation in DNAm. We used the *sva* package with default parameters to estimate surrogate variables [18].

# References

Gervin K, Nordeng H, Ystrom E, Reichborn-kjennerud T, Lyle R. Long-term prenatal exposure to paracetamol is associated with DNA methylation differences in children diagnosed with ADHD. Clin Epigenetics. 2017;9.

World Health Organization. Chapter V: Mental and Behavioural Disorders. The 10^th^ revision of the International Statistical Classification of Diseases and Related Health Problems. Geneva; 2018.

American Psychiatric Association. Neurodevelopmental Disorders. The Diagnostic and Statistical Manual of Mental Disorders. American Psychiatric Association; 2013.

Thapar A, Pine DS, Leckman JF, Scott S, Snowling MJ, Taylor E. Rutter’s Child and Adolescent Psychiatry: Sixth Edition [Internet]. Rutter’s Child Adolesc. Psychiatry Sixth Ed. Chichester; 2015.

HM Wieselberg, M Rutter, G Thorley. Which boys respond to stimulant medication? A controlled trial of methylphenidate in boys with disruptive behaviour. Psychol Med. 1987;121–43.

Roth C, Bjørke-Monsen AL, Reichborn-Kjennerud T, Nilsen RM, Smith GD, Stoltenberg C, et al. Use of folic acid supplements in early pregnancy in relation to maternal plasma levels in week 18 of pregnancy. Mol Nutr Food Res. 2013;57:653–60.

Bjørke-Monsen AL, Roth C, Magnus P, Midttun Ø, Nilsen RM, Reichborn-Kjennerud T, et al. Maternal B vitamin status in pregnancy week 18 according to reported use of folic acid supplements. Mol Nutr Food Res. 2013;57:645–52.

Olstad EW, Nordeng HME, Gervin K. Prenatal medication exposure and epigenetic outcomes: a systematic literature review and recommendations for prenatal pharmacoepigenetic studies. Epigenetics. 2021.

Fages A, Ferrari P, Monni S, Dossus L, Floegel A, Mode N, et al. Investigating sources of variability in metabolomic data in the EPIC study: the Principal Component Partial R-square (PC-PR2) method. Metabolomics. 2014;10:1074–83.

Perrier F, Novoloaca A, Ambatipudi S, Baglietto L, Ghantous A, Perduca V, et al. Identifying and correcting epigenetics measurements for systematic sources of variation. Clin Epigenetics. 2018;10.

Aryee MJ, Jaffe AE, Corrada-Bravo H, Ladd-Acosta C, Feinberg AP, Hansen KD, et al. Minfi: a flexible and comprehensive Bioconductor package for the analysis of Infinium DNA methylation microarrays. 2014;30:1363–9.

Houseman EA, Molitor J, Marsit CJ. Reference-free cell mixture adjustments in analysis of DNA methylation data. Bioinformatics. 2014;30:1431–9.

Gervin K, Page CM, Aass HCD, Jansen MA, Fjeldstad HE, Andreassen BK, et al. Cell type specific DNA methylation in cord blood: A 450K-reference data set and cell count-based validation of estimated cell type composition. Epigenetics. 2016;11:690–8.

Gervin K, Salas LA, Bakulski KM, Van Zelm MC, Koestler DC, Wiencke JK, et al. Systematic evaluation and validation of reference and library selection methods for deconvolution of cord blood DNA methylation data. Clin Epigenetics. 2019;11.

Du P, Zhang X, Huang C-C, Jafari N, Kibbe WA, Hou L, et al. Comparison of Beta-value and M-value methods for quantifying methylation levels by microarray analysis. BMC Bioinformatics. 2010;11:587.

Smyth GK. limma: Linear Models for Microarray Data. Bioinforma Comput Biol Solut Using R Bioconductor. 2005. p. 397–420.

Benjamini Y. Discovering the false discovery rate. J R Stat Soc Ser B. 2010;72:405–16.

Leek JT, Johnson WE, Parker HS, Jaffe AE, Storey JD. The SVA package for removing batch effects and other unwanted variation in high-throughput experiments. Bioinformatics. 2012;28:882–3.

Bateman BT, Mhyre JM, Hernandez-Diaz S, Huybrechts KF, Fischer MA, Creanga AA, et al. Development of a comorbidity index for use in obstetric patients. Obstet Gynecol. 2013;122:957–65.

Easter SR, Bateman BT, Sweeney VH, Manganaro K, Lassey SC, Gagne JJ, et al. A comorbidity-based screening tool to predict severe maternal morbidity at the time of delivery. Am J Obstet Gynecol. 2019;221:271.e1-271.e10.

# Supplementary Figures

***Figure S1.*** *Principal component analysis (PCA) was used to compute the principal components (PCs), which represent DNA methylation variation.* ***(A)*** *The significance of the association between the top three PCs and various covariates (association tests included the Spearman’s correlation test [continuous variables] and one-way analysis of variance [ANOVA; categorical variables]).* ***(B)*** *Scree plot indicating the contribution to DNAm variation by each of the 12 first PCs.* ***(C)*** *The weighted partial R^2^ for each covariate (i.e., the variability contributed by the respective covariate to the top three PCs, when accounting for the variability contribution of all other covariates in the model).* ***Abbreviations:*** *BMI: body mass index; nRBC: nucleated red blood cell; NSAID: non-steroidal anti-inflammatory drug; Tech.: technical covariate.*

**
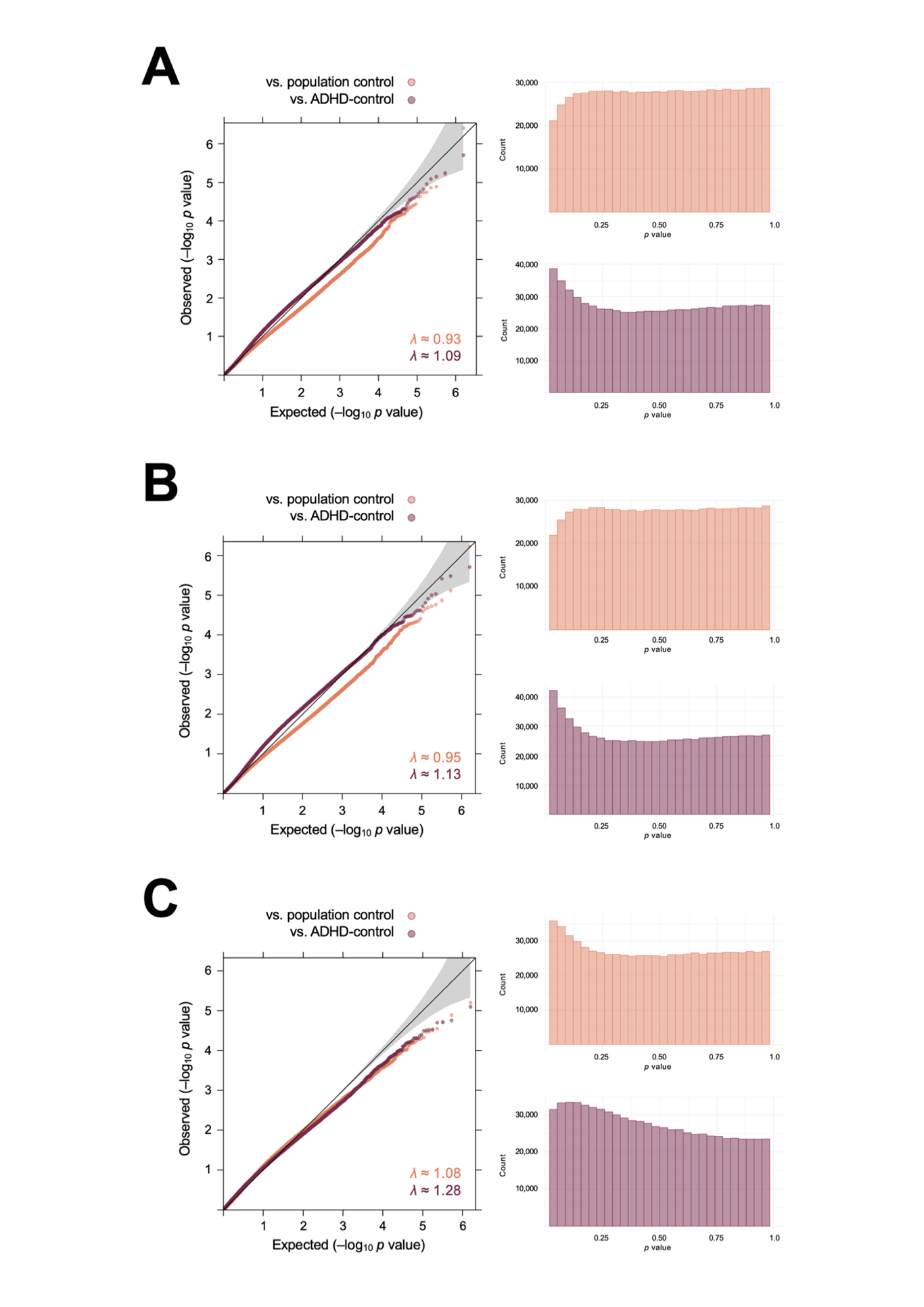
**

***Figure S2.*** *Quantile-quantile (QQ) plots and histograms of the* p *values of* ***(A)*** *the crude model,* ***(B)*** *the adjusted model, and* ***(C)*** *the interaction model,* not *adjusted for the CD8^+^ T cell proportion. The* p *values reflect the pairwise comparisons of the exposed group to the population control group (orange) or the ADHD-control group (purple).* λ *is the genomic inflation factor.*

**
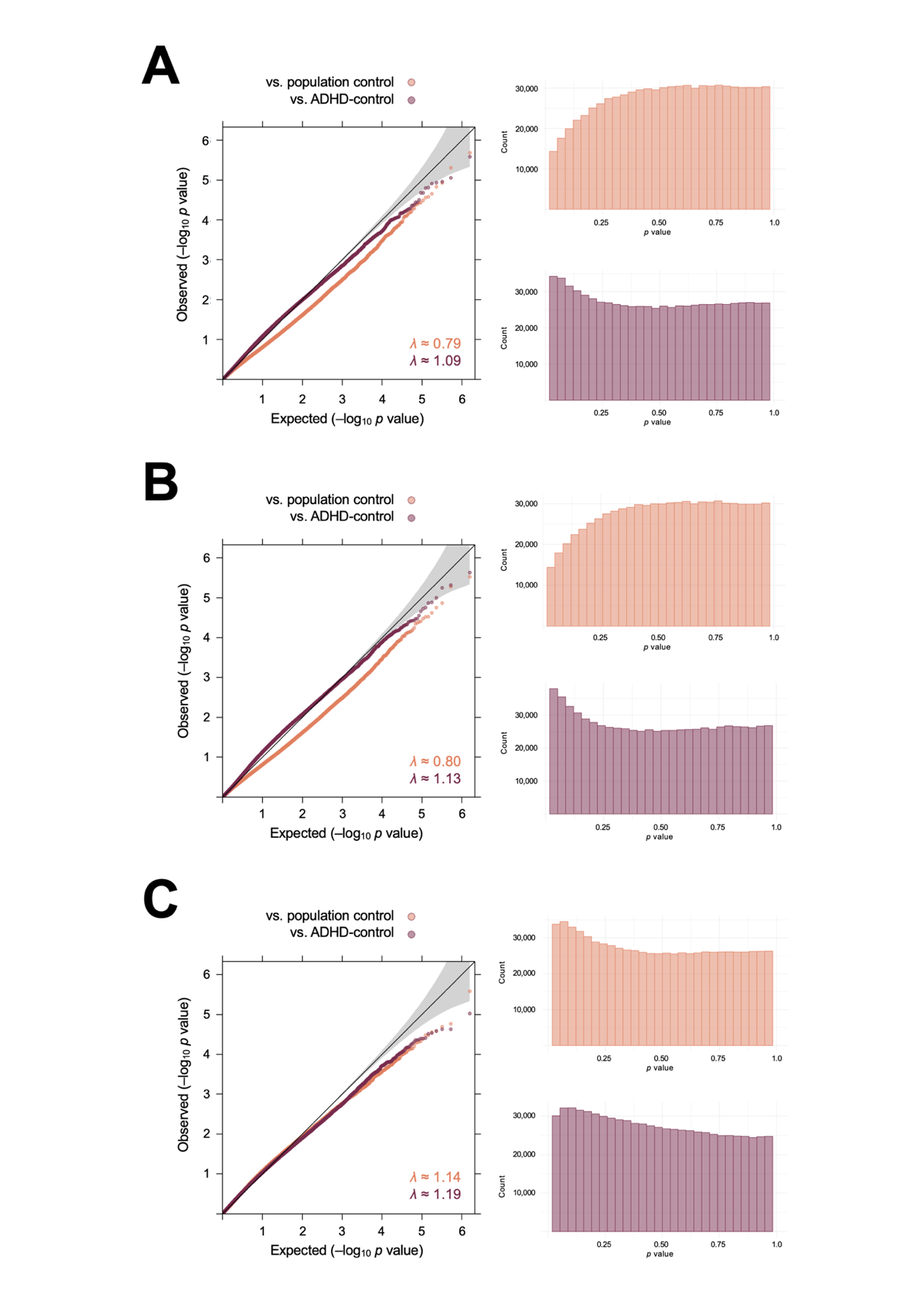
**

***Figure S3.***  *Quantile-quantile (QQ) plots and histograms of the* p *values of* ***(A)*** *the crude model,* ***(B)*** *the adjusted model, and* ***(C)*** *the interaction model, all adjusted for the CD8^+^ T cell proportion. The* p *values reflect the pairwise comparisons of the exposed group to the population control group (orange) or the ADHD-control group (purple).* λ *is the genomic inflation factor.*

# Supplementary Tables

**Table S1.** Overview of the comparison group characteristics stratified by folic acid status. *P* values for significant differences between the exposed group and the two control groups, were calculated using the Wilcoxon rank-sum test (continuous variables), and the Chi-squared test or Fisher’s exact test (categorical variables).

|  | | **Exposed**  (*n* = 61) | | | **ADHD-control**  (*n* = 100) | | | | | | **Population control**  (*n* = 100) | | | | ***p*** |
| --- | --- | --- | --- | --- | --- | --- | --- | --- | --- | --- | --- | --- | --- | --- | --- |
|  | | **Folic acid**  (*n* = 37) | | **No folic acid**  (*n* = 24) | | **Folic acid**  (*n* = 45) | | **No folic acid**  (*n* = 55) | | **Folic acid**  (*n* = 52) | | | **No folic acid**  (*n* = 48) | |  |
| **Maternal characteristics** | | | | | | | | | | | | | | | |
| **Maternal age** (mean years ± SD) | 31.0 ± 4.5 | | 28.8 ± 4.7 | | 29.6 ± 4.6 | | 27.8 ± 5.2 | | 30.2 ± 5.1 | | | 29.2 ± 5.3 | | **N.S.** | |
| **Pre-pregnancy BMI** (mean BMI ± SD) | 27.0 ± 5.9 | | 26.8 ± 5.1  *3 NA* | | 25.2 ± 4.4  *1 NA* | | 24.9 ± 5.3  *3 NA* | | 23.2 ± 3.7  *1 NA* | | | 24.1 ± 3.6  *2 NA* | | **^a,b^** | |
| **Primiparous** (yes; *n* (%)) | 10 (27.0) | | 8 (33.3) | | 27 (60.0) | | 22 (40) | | 27 (51.9) | | | 21 (43.8) | | **^c,d^** | |
| **Married or cohabiting** (yes; *n* (%)) | 16 (43.2) | | 13 (54.2) | | 16 (35.6) | | 23 (41.8) | | 27 (51.9) | | | 21 (43.8) | | **N.S.** | |
| **Maternal education**  University/college (*n* (%))  High school or lower (*n* (%)) | 13 (35.1)  24 (64.9) | | 12 (50.0)  11 (45.8)  *1 NA* | | 24 (53.3)  19 (42.2)  *2 NA* | | 17 (30.9)  37 (67.3)  *1 NA* | | 35 (67.3)  15 (28.8)  *2 NA* | | | 23 (47.9)  21 (43.8)  *4 NA* | | **^e^** | |
| **Smoking in pregnancy** (yes; *n* (%)) | 6 (16.2) | | 6 (25.0) | | 2 (4.4) | | 11 (20.0) | | 1 (1.9) | | | 6 (12.5) | | **^f^** | |
| **Alcohol in pregnancy**  (yes; *n* (%)) | 8 (21.6) | | 3 (12.5)  *1 NA* | | 9 (20.0) | | 16 (29.1)  *1 NA* | | 10 (19.2) | | | 16 (33.3)  *1 NA* | | **N.S.** | |
| **Multivitamins in pregnancy** (yes; *n* (%)) | 18 (48.6) | | 5 (20.8) | | 25 (55.6) | | 9 (16.4) | | 36 (69.2) | | | 9 (18.8) | | **N.S.** | |
| **Maternal medications*** | | | | | | | | | | | | | | | |
| **Days of paracetamol** (median days; 1^st^–3^rd^ quartile) | 30.0 (25.0–60.0) | | 27.5 (21.0–37.0) | | --- | | --- | | --- | | | --- | | **---** | |
| **Psychotropics**** (yes; *n* (%)) | 8 (21.6) | | 3 (12.5) | | 5 (11.1) | | 4 (7.3) | | 0 (0) | | | 3 (6.3) | | **^g^** | |
| **NSAIDs** (yes; *n* (%)) | 13 (35.1) | | 9 (37.5) | | 1 (2.2) | | 9 (16.4) | | 4 (7.7) | | | 3 (6.3) | | **^h,i^** | |
| **Other analgesics***** (yes; *n* (%)) | 7 (18.9) | | 10 (41.7) | | 2 (4.4) | | 1 (1.8) | | 2 (3.9) | | | 3 (6.3) | | **^j,k^** | |
| **Maternal morbidities** | | | | | | | | | | | | | | | |
| **Comorbidity index****** (mean score ± SD) | 0.8 ± 1.6 | | 0.9 ± 1.0  *3 NA* | | 0.6 ± 1.2  *1 NA* | | 0.6 ± 1.1  *2 NA* | | 0.4 ± 1.0  *3 NA* | | | 0.5 ± 1.0  *1 NA* | | **N.S.** | |
| **Chronic diseases*******  None (*n* (%))  1–2 diseases (*n* (%))  ≥3 diseases (*n* (%)) | 32 (86.5)  5 (13.5)  0 (0) | | 22 (91.7)  2 (8.3)  0 (0) | | 43 (95.6)  2 (4.4)  0 (0) | | 49 (89.1)  6 (10.9)  0 (0) | | 52 (100)  0 (0)  0 (0) | | | 45 (93.8)  3 (6.2)  0 (0) | | **^l^** | |
| **Conception and birth** | | | | | | | | | | | | | | | |
| ***In vitro* fertilisation** (yes; *n* (%)) | 0 (0) | | 0 (0) | | 3 (6.7) | | 0 (0) | | 2 (3.8) | | | 1 (2.1) | | **N.S.** | |
| **Caesarean section** (yes; *n* (%)) | 2 (5.4) | | 5 (20.8) | | 6 (13.3) | | 9 (16.4) | | 7 (13.5) | | | 5 (10.4) | | **N.S.** | |
| **Child characteristics** | | | | | | | | | | | | | | | |
| **Gestational age at birth** (mean weeks ± SD) | 39.6 ± 1.4  *1 NA* | | 39.2 ± 1.4 | | 39.1 ± 1.9 | | 39.5 ± 1.6 | | 39.7 ± 1.7 | | | 39.5 ± 1.6 | | **N.S.** | |
| **Infant sex** (female; *n* (%)) | 14 (37.8) | | 11 (45.8) | | 14 (31.1) | | 12 (21.8) | | 25 (48.1) | | | 19 (39.6) | | **N.S.** | |
| **Birth weight** (mean grams ± SD) | 3,601 ± 456 | | 3,564 ± 376 | | 3,468 ± 523 | | 3,672 ± 540 | | 3,603 ± 463 | | | 3,495 ± 583 | | **N.S.** | |
| **Technical covariates** | | | | | | | | | | | | | | | |
| **Bisulfite conversion**  Plate 1 (*n* (%))  Plate 2 (*n* (%))  Plate 3 (*n* (%)) | 16 (0.43)  12 (0.32)  9 (0.24) | | 11 (0.46)  9 (0.38)  4 (0.17) | | 16 (0.36)  20 (0.44)  9 (0.20) | | 14 (0.26)  28 (0.51)  13 (0.24) | | 20 (0.39)  14 (0.27)  18 (0.35) | | | 19 (0.40)  13 (0.27)  16 (0.33) | | **N.S.** | |

**Abbreviations:** ADHD: attention-deficit/hyperactivity disorder; BMI: body mass index; NA: missing value; N.S.: not significant; SD: standard deviation.

* Within-group percentages may add up to more than 100% as one woman may have used medications from several medication groups.

** Includes all medications with the N02 Anatomical Therapeutic Chemical (ATC) code except paracetamol (i.e., opioids, antimigraine preparations, and other analgesics and antipyretics)

*** Includes all antidepressants, antiepileptics and antipsychotics.

**** Includes all variables available in MBRN and MoBa from a list provided in Bateman *et al.* (2013) [19, 20]. The different variables are given different weights (weight in parentheses).The variables included in the final score are: asthma (1), cardiovascular disease (3), chronic renal disease (1), congenital heart disease (4), illicit substance use (2), gestational hypertension (1), mild-unspecified preeclampsia (2), severe preeclampsia (5), placenta previa (2), pre-existing diabetes mellitus (1), pre-existing hypertension (1), previous Caesarean delivery (1), lupus (2), alcohol abuse (weekly consumption; 1) and maternal age group (> 44 years: 3; 40–45 years: 2; 35–40 years: 1; < 35 years: 0).

***** Chronic diseases included were asthma, rheumatoid arthritis, epilepsy, Crohn’s disease, lupus, multiple schlerosis (MS), cancer and diabetes mellitus. All diseases were weighted equally and each additional disease added 1 to the final score.

^a^ *p* ≈ 0.04, comparing exposed to ADHD-controls

^b^ *p* < 0.0001, comparing exposed to population controls

^c^ *p* ≈ 0.02, comparing exposed to ADHD-controls

^d^ *p* ≈ 0.03, comparing exposed to population controls

^e^ *p* ≈ 0.02, comparing exposed to population controls

^f^ *p* ≈ 0.03, comparing exposed to population controls

^g^ *p* ≈ 0.01, comparing exposed to population controls

^h^ *p* < 0.0001, comparing exposed to ADHD-controls

^i^ *p* < 0.0001, comparing exposed to population controls

^j^ *p* < 0.0001, comparing exposed to ADHD-controls

^k^ *p* < 0.0001, comparing exposed to population controls

^l^ *p* ≈ 0.05, comparing exposed to population controls

**Table S2.** Overview of the cell type composition of the comparison groups stratified by folic acid status. *P* values for significant differences between the exposed group and the two control groups were calculated using the Wilcoxon rank-sum test and any significant comparisons are detailed in the caption.

|  | **Exposed**  (*n* = 61) | | **ADHD-control**  (*n* = 100) | | **Population control**  (*n* = 100) | | ***p*** |
| --- | --- | --- | --- | --- | --- | --- | --- |
|  | **Folic acid**  (*n* = 37) | **No folic acid**  (*n* = 24) | **Folic acid**  (*n* = 45) | **No folic acid**  (*n* = 55) | **Folic acid**  (*n* = 52) | **No folic acid**  (*n* = 48) |  |
| **B cells;**  *mean proportion ± s.d.* | 0.03 ± 0.02 | 0.03 ± 0.02 | 0.03 ± 0.02 | 0.03 ± 0.02 | 0.03 ± 0.02 | 0.03 ± 0.02 | **N.S.** |
| **CD4^+^ T cells;**  *mean proportion ± s.d.* | 0.14 ± 0.06 | 0.16 ± 0.05 | 0.15 ± 0.06 | 0.15 ± 0.06 | 0.14 ± 0.05 | 0.14 ± 0.06 | **N.S.** |
| **CD8^+^ T cells;**  *mean proportion ± s.d.* | 0.04 ± 0.03 | 0.04 ± 0.02 | 0.04 ± 0.02 | 0.04 ± 0.02 | 0.03 ± 0.02 | 0.03 ± 0.02 | **^a^** |
| **Granulocytes;**  *mean proportion ± s.d.* | 0.57 ± 0.10 | 0.55 ± 0.09 | 0.57 ± 0.11 | 0.58 ± 0.09 | 0.59 ± 0.10 | 0.58 ± 0.09 | **N.S.** |
| **Monocytes;**  *mean proportion ± s.d.* | 0.06 ± 0.04 | 0.05 ± 0.03 | 0.05 ± 0.03 | 0.05 ± 0.03 | 0.06 ± 0.04 | 0.05 ± 0.04 | **N.S.** |
| **Natural killer cells;** *mean proportion ± s.d.* | 0.02 ± 0.02 | 0.02 ± 0.02 | 0.02 ± 0.02 | 0.01 ± 0.02 | 0.02 ± 0.02 | 0.02 ± 0.03 | **N.S.** |
| **nRBCs;**  *mean proportion ± s.d.* | 0.12 ± 0.10 | 0.14 ± 0.10 | 0.14 ± 0.12 | 0.12 ± 0.10 | 0.12 ± 0.08 | 0.11 ± 0.09 | **N.S.** |

**Abbreviations:** ADHD: attention-deficit hyperactivity disorder; nRBC: nucleated red blood cell; N.S.: not significant.

^a^ *p* ≈ 0.03, exposed to population controls

**Table S3.** List of the CpGs’ (FDR-adjusted) *p* values for the crude, adjusted and interaction models.

*Please find the table uploaded on figshare (doi:* [*10.6084/m9.figshare.23338874*](https://figshare.com/articles/journal_contribution/Table_S3_xlsx/23338874)*).*

**Table S4.** List of the CpGs’ (FDR-adjusted) *p* values when running the crude model with preprocessing and modelling similar to our previous study [1]. We ran three different crude models: (1) using DNAm data normalized with noob and BMIQ, (2) adjusting for the same covariates as in our previous study, and (3) using both noob/BMIQ-normalized data and adjusting for these covariates.

*Please find the table uploaded on figshare (doi:* [*10.6084/m9.figshare.23338871*](https://figshare.com/articles/journal_contribution/Table_S4_xlsx/23338871)*).*

**Table S5.** List of the CpGs’ (FDR-adjusted) *p* values when running the crude model only with the probes overlapping between the 450k and EPIC microarray platforms (*n* = 397,834 CpGs).

*Please find the table uploaded on figshare (doi:* [*10.6084/m9.figshare.23338868*](https://figshare.com/articles/journal_contribution/Table_S5_xlsx/23338868)*).*
